# Efficacy of mucolytics and steam therapy in the management of sinusitis among Indians

**DOI:** 10.6026/97320630019479

**Published:** 2023-04-30

**Authors:** Rajalakshmi S Balakrishnan, Subhashree Rohinikumar, Abhinav Rajendra Prabhu, Vishnu Priya Veeraraghavan, Thiyaneswaran Nesappan, Rajalakshmanan Easwaramoorthy

**Affiliations:** 1 Department of Implantology, Saveetha Dental College and Hospitals, Saveetha Institute of Medical and Technical Science, Chennai- 600077, India; 2 Department of Biomaterials, Centre of Molecular Medicine and Diagnostics (COMManD), Saveetha Dental College and Hospitals, Saveetha Institute of Medical and Medical and Technical Sciences, Saveetha University, Chennai 600077, India

**Keywords:** Sinus membrane thickening, maxillary ostium, mucolytics, steam therapy

## Abstract

It is of interest to compare the treatment modalities of sinus membrane thickening, by analyzing the difference in pre and post-intervention radiographic measurement of sinus membrane thickness. Results showed that combination therapy of steam and mucolytics decreased the sinus thickening in a statistically significant manner compared to mucolytics alone. Thus, there is a correlation between maxillary sinus membrane thickening and patency of maxillary ostium radiographically.

## Background:

The maxillary sinus, first discovered by Leonardo Da Vinci in the year 1498, is the first of all paranasal air sinuses to develop [1]. It consists of apex that is pointed towards the zygomatic process, base that is anatomically related to the lateral wall of nasal cavity, and has orbital floor as the superior wall, maxillary alveolar process as the inferior wall, pterygopalatine fossa as posterior wall and maxillary facial surface as anterior wall. The vascular supply of the maxillary sinus is from greater palatine artery, infraorbital artery and facial artery. The venous drainage is through the pterygoid plexus of veins, sphenopalatine and facial vein. The maxillary sinus, first discovered by Leonardo Da Vinci in the year 1498, is the first of all paranasal air sinuses to develop [1]. It consists of apex that is pointed towards the zygomatic process, base that is anatomically related to the lateral wall of the nasal cavity, and has orbital floor as the superior wall, maxillary alveolar process as the inferior wall, pterygopalatine fossa as the posterior wall and maxillary facial surface as anterior wall. The vascular supply of the maxillary sinus is from greater palatine artery, infraorbital artery and facial artery. The venous drainage is through the pterygoid plexus of veins, sphenopalatine and facial vein [2]. The maxillary sinus drains through the ostium. The medial wall of the maxillary sinus houses the maxillary ostium in its superior aspect, just beneath the floor of the orbit. Researchers have found that the distance between the floor of the sinus and the maxillary ostium is around 29 mm [3]. The diameter of the maxillary sinus ostium is around 3-10mm and the shape of the ostium is slit shaped or oval shaped and is directed either obliquely or horizontally [3]. An important clinical and radiographically significant structure is the ostiomeatal complex consisting of the maxillary ostium, frontal recess and anterior cells of ethmoid and ethmoid infundibulum. The pathway of the drainage initiates from the ostium followed by drainage into the narrow infundibulum, finally draining into the meatus [4]. When this channel is blocked, rhinosinusitis may occur leading to postoperative complications after implant placement and augmentation procedures. Thus identification of the ostiomeatal complex, followed by assessment of the anatomy and pathological condition that leads to increased sinus mucosal thickness in the radiographic image, is an important phase in the diagnosis and treatment planning, to avoid the postoperative complications [5]. Several imaging modalities have been in use in the novel field of dentistry to visualize the maxillary sinus and its associated structures , some of which include Water’s/paranasal sinus view, Computed Tomography(CT),Magnetic Resonance Imaging (MRI)cone-beam computed tomography (CBCT), among which the gold standard till date is CT imaging[6]. However due to high exposure to radiation and high expense with respect to CT imaging modality, CBCT serves to be a promising alternative to appreciate the sinus opacifications and other inflammatory radiographic appearances with less exposure [7]. Therefore, it is of interest to find the incidence of sinus ostium blockade in cases of sinus membrane thickening of >2mm in CBCT imaging modality among the South Indian Population.

## Materials and Methods:

## CBCT analysis for prevalence of the maxillary ostium blockade:

130 Full skull CBCT of CS 3D Software were analyzed in the maxillary posterior region and correlation between maxillary sinus ostium opening and the maxillary sinus membrane thickening were analyzed. The observations were done by two observers and cross verified. The collected data was statistically analyzed and the data was tabulated.

## Patient selection for comparing treatment modalities:

The patients whose age was above 18 years with either acute symptoms of sinonasal pathology or with chronic and recurrent sinusitis were chosen for the study. Subjects were enrolled in the month of July, 2022 at Saveetha Dental College, Chennai. . All patients were examined by an experienced implantologist according to a standardized clinical procedure, the same day as the CBCT was performed.

The inclusion criteria are as follows:

[1] Presence of an edentulous region in the posterior region of the maxilla

[2] Sinus lining thickening of > 5mm

[3]≥18 years of age

[4] Systemically healthy

[5] Non-pregnant.

The exclusion criteria are as follows:

[1] Pregnant and lactating mothers

[2] (ii)Systemically compromised patients with chronic liver and chronic kidney disease

[3] (iii)Patients under 18 years of age.

Clinally, 30 patients who had sinus membrane thickening were divided into two groups. The first group(n=15) was treated with mucolytics (Mucolite 30 mg) alone, the second group was treated with mucolytics and steam therapy for about 20 minutes twice a day for about 10 days. The CBCT was retaken for all the patients after 10 days and the results were recorded and statistics were performed using IBM SPSS software version 2.0.

## Results:

## Prevalence of sinus membrane thickness and ostium blockade:

Out of the observed 130 full skull CBCT, 42 patients had sinus membrane thickening and 31 patients with sinus membrane thickening had sinus maxillary ostium blockade.

## Statistical results for comparison of sinus membrane thickness treatment modalities:

Prevalence of sinus membrane thickening = 42/130 = 32.3% Prevalence of sinus membrane thickening with ostium blockage = 31/42 = 73% 

**Figure 1:**
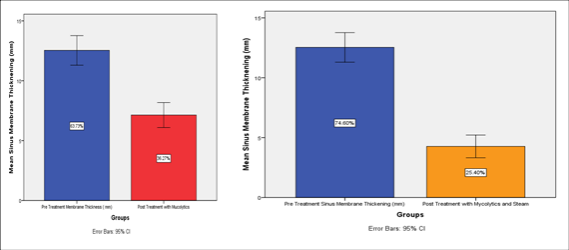
Represents the graphical comparison depicting the difference in the measured length pre and post and intervention in the individual treatment groups.

The CBCT were taken after 10 days and the thickness of the sinus membrane was measured at the previously measured site and the length measured pre and post treatments were tabulated and the statistical results were obtained and compared using SPSS software 2.0. Independant sample -t test was used to compare the difference in sinus membrane thickening before and after the intervention in both the groups (Table 1 and 2). The statistical tests revealed that both the groups significantly reduced the sinus membrane thickening after intervention (Figure 1). However, it is more pronounced in the experimental group where the combination of mucolytics and steam therapy was given.

## Discussion:

Due to the maxillary sinus expansion primarily caused by remodelling of the bone and pneumatisation process, the recent trend has been shifted to augment the available residual bone height for successful implant placement by direct and indirect methods [8]. The extraction of the maxillary molar tooth is followed by marked maxillary sinus expansion of about 1-5 mm in a period of one year [9]. Also the distance between the root apices of the maxillary molar and the maxillary sinus floor has been observed to physiologically decrease with increasing age [10] . Several systematic reviews have put forth the comparison of survival rate of implants that were placed in augmented sinus floor sites and the survival rate of short implants [11]. According to Corbella *et al*. 86.5% to 98.2% was the survival rate of short implants, 95.4 to 100% was the survival rate of implants placed through indirect sinus lift technique and 75.5 to 100% was the survival rate of the implant placed through direct sinus lift technique [12]. Thus due to high reliability, several implant companies have been competing to patent newer techniques that increase the ease of sinus lifting procedure [13].

Histologically, the Schneiderian mucous membrane contains epithelium that is pseudo stratified ciliated and connective tissue that is highly vascularised in nature [14]. Kim *et al*. in 2009 proved the incredible capacity of the Schneiderian membrane's mesenchymal cells to form bone, playing a pivotal role in sinus augmentation procedures. Till date, the membrane thickness have been assessed using CBCTs, CTs and in cadavers and the mean thickness was found to be 1.60 ± 1.20mm [15]. However, it is most common to see sinus membrane thickening even in patients who are clinically asymptomatic and it is concluded that sinus membrane thickness of more than 4 mm is pathological in nature [16]. It is highly essential for the clinician to correlate the radiographic and clinical findings and plan the treatment accordingly [17]. The cause of the sinus membrane thickening and fluid accumulation may be due to "Acute rhinosinusitis" if the symptoms such as posterior or anterior nasal discharge, blockage of nose, congestion develops within 12 weeks of time duration [18] .Radiographic imaging reveals changes in the mucosa with hazy, fluid filled ostiomeatal complex. Endoscopic examination reveals middle meatus discharge that is mucopurulent in nature obstructing the middle meatus [19]. Initially, several controversies existed regarding the origin of rhinosinusitis - whether it is bacterial or viral, however, studies have proved that it is commonly viral in origin that develops into bacterial rhino sinusitis [20] . The symptoms of acute rhinosinusitis peak between 3-4th day and gradually alleviates within 11-14 days [21]. It is associated with nasal discharge that is mucopurulent in nature corresponding to the neutrophil inflammatory infiltrate that does not necessarily represent infection from bacteria. The treatment of the acute rhinosinusitis is mainly directed on providing symptomatic relief such as analgesics, steam inhalation, mucolytics, anti-inflammatory drugs, anti-histamines, steroids, nasal saline irrigation. The most common pathogenic bacteria associated with acute rhinosinusitis in decreasing order of incidence are *Haemophilus influenzae*, *Moraxella catarrhal*, *Streptococcus pneumoniae* and *Staphylococcus aureus*. The first line of treatment includes amoxicillin (oral twice daily for 90mg/kg) or amoxicillin-clavulanate. In patients allergic to penicillin (non-type 1), clindamycin or third- generation cephalosporin (cefpodoxime or cefixme) is indicated [22]. If the symptoms are present for more than 12 weeks, it is suggestive of chronic-rhinosinusitis. In addition to penicillins and cephalosporins, aminoglycosides fluoroquinolones, corticosteroids are other drugs used in the treatment of rhinosinusitis. To help in the regeneration of the mucous membrane, irrigation of the nasal cavity using saline has been proven to improve the movement of mucociliary pathway, eliminate antigenic agents, inflammatory infiltrates and biofilms to ultimately protect and promote the health of mucous membrane [23]. However, chronic polypoid lesions may take 3-6 months to completely resolve.

Steam inhalation therapy has been proven to have an appreciable impact on viral load, alteration of host defence mechanism, activation of adaptable thermo-modulatory mechanism that enables to reinstate homeostasis .The heat directly inhibits the pathogens, enhances both the innate and acquired immunity. The inhaled steam, by creating a stress to the lungs, increases the airflow, vital capacity, forced expiratory lung volume, thus efficiently reducing the symptoms of rhinosinusitis and nasal congestion [24]. The steam therapy is practiced as an adjunct to treat respiratory pathologies as a broncholite and a study done by Lowen and Steel has proven that the mucositis causing virus such as influenza virus, SARS-CoV-2 could be inactivated invitro and invivo when it is exposed to a temperature beyond 30 degree Celsius [25]. The alteration in the pH of the blood induced by hyperthermia results in transient pulmonary alkalosis is known. Since the majority of mucosinusitis causing pathogens shows maximum activity in an acidic environment, the multiplication, replication and mobilization rate can be suppressed in the steam induced alkaline condition [25]. According to Sturman *et al*. the overall effectiveness of steam inhalation includes activation of parasympathetic activity, decrease in blood pressure, decrease in inflammation, decreased oxidative stress, increased cardiac output, increased plasma volume, increased peripheral blood flow and enhances endothelial function and reinforces the arterial function.

The steam inhalation induces several cellular mechanisms, including activation of the immune system, release of heat shock proteins (HSP). HSP functions as chaperones that are released into the bilayer lipid wall of the cells to withstand the temperature. Steam inhalation therapy has been insisted upon from the ancestral period as a natural treatment for sinusitis. Inhaling steam has been proven to break and clear the mucus, which in turn can improve the ease of breathing and can potentially reduce the symptoms of the mucosal inflammation. The above discussion is in relevance with the results of the current study where the steam inhalation has enhanced various mechanisms of action of the body's defense pathways against the pathogenic microorganisms. The decrease in sinus inflammation was radiographically confirmed with the CBCT values, pre and post intervention which was found to be statistically signification on further evaluation.

## Conclusion:

Data shows that there is correlation between maxillary sinus membrane thickening and patency of maxillary ostium. Further the combination of steam and mucolytics treatment decreased the sinus thickening in a statistically significant manner compared to mucolytics alone .Both the treatments can be reliably given for maxillary sinus membrane thickening before sinus lift procedures.

## Figures and Tables

**Table 1:** Represents the results of independent sample T test for measurements obtained pre and post treatment with mucolytics and steam.

**GROUPS**	**N**	**MEAN**	**STD.DEVIATION**	**STD.ERROR MEAN**
Pre treatment sinus membrane thickening (mm)	15	12.53	2.232	0.576
Post treatment with Mucolytics and steam	15	4.27	1.71	0.441
**INDEPENDENT SAMPLE TEST**									
	**Levene's Test for equaltity of variances**		**t-test for equality of means**					**95% Confidence interval if the deifferences**	
	**F**	**Sig**	**t**	dt	**Sig(2 tailed)**	**Mean Difference**	**Std.Error Difference**	**LOWER**	**UPPER**
Equal variances assumed	1.622	0.213	11.388	28	0	8.267	0.726	6.78	9.754
Equal variances not assumed			11.388	26.224	0	8.267	0.726	6.775	9.758

**Table 2:** Represents the results of independent sample t test for measurements obtained pre and post treatment with mucolytics alone

**GROUPS**	**N**	**MEAN**	**STD.DEVIATION**	**STD.ERROR MEAN**
Pre treatment Sinus membrane thickening (mm)	15	12.53	2.232	0.576
Post treatment with Mucolytics alone(mm)	15	7.13	1.895	0.487
**INDEPENDENT SAMPLE TEST**									
	**Levene's Test for equaltity of variances**		**t-test for equality of means**					**95% Confidence interval if the deifferences**	
	**F**	**Sig**	**t**	**dt**	**Sig(2 tailed)**	**Mean Difference**	**Std.Error Difference**	**LOWER**	**UPPER**
Equal variances assumed	0.727		7.159	28	0	5.4	0.754	3.855	6.945
Equal variances not assumed		0.401	7.159	27.234	0	5.4	0.754	3.853	6.947
